# Book Review

**Published:** 1941

**Authors:** 


### Reviews of Books

**Syphilis in Earlier Days.** By J. R. Whitwell, M.B. Pp. viii., 89. Illustrated. London: H. K. Lewis & Co. Ltd. 1940. Price 5s.—This not very interesting book endeavours by means of many quotations from ancient authors to prove that syphilis was not a new disease introduced from the New World by the crews of the early navigators and in particular of Columbus. The quotations are mostly such vague descriptions of ailments that a great deal can be read into the symptoms enumerated. The Old Testament provides typically inexact statements which Dr. Whitwell brings forward in support of his thesis. The author is more interesting when he gives a survey in Chapter vi of the steps by which General Paralysis was first recognized as an entity and then correctly attributed to syphilis. Even here there is a remarkable omission of any reference to the work of Mott.

**The Chronicle of Crichton Royal, 1833–1936.** By C. C. Easterbrook, M.D., F.R.C.P. Pp. xii., 663. Illustrated. Dumfries: Courier Press. 1940.—This handsome volume summarizes with the aid of annual reports the achievements of a century in the history of one of the earliest and most progressive mental hospitals in the country. The statistics and the accounts of how institutional problems have been overcome are of special value and the ordinary reader will find much of interest in this record of the development of an enlightened attitude towards insanity. A clinical summary compiled from the case material of the hospital expresses well-considered views on the causation, prognosis, and treatment of mental illnesses. This book is beautifully produced in clear type with numerous illustrations.

**Text-Book of Medicine.** Fifth Edition. Edited by J. J. Conybeare, M.C., D.M., F.R.C.P. Pp. xx., 1131. Illustrated. Edinburgh: E. & S. Livingstone. 1940. Price 24s.—The rapid appearance of a fifth edition of this text-book testifies to its value and popularity. Readers will find the cardio-vascular section has been practically re-written, especially the portion dealing with "Essential Hypertension." It is regrettable that this meaningless adjective "essential" is retained; the definition that it means an "apparently primary condition" does not excuse the term "essential." However, the advice given for dealing with cases of unexplained hypertension is excellent. "Chemotherapy," particularly in relation to sulphonamides, is briefly and lucidly described, and the necessity for blood counts is emphasized. "Blood transfusion" has been largely re-written and is a good feature of the book. In "Diseases of the Alimentary Tract" it is pleasing to find that gastroscopy receives the attention which is deserves. Conybeare's "Text-Book of Medicine" is one of the very best compact text-books for practitioners and students. The publisher's share in the production deserves high praise, the type and the paper are easy to the eye, and the skiagrams are beautifully reproduced. To those who know Mr. Marxer's work perhaps it is not surprising that his skiagrams should reproduce well.

**A Practical Manual of Diseases of the Chest.** Second Edition. By MAURICE DAVIDSON, M.D., F.R.C.P. Pp. xiv., 575. Illustrated. London: Oxford University Press (Humphrey Milford). 1941. Price 42s.—It is pleasing to see another edition of this book on chest diseases. Both from the point of view of production and subject matter it is probably the best English text-book on pulmonary diseases. It is essentially practical in outlook, and the clinician will find all the major problems of lung disease clearly discussed. The illustrations, particularly skiagrams, are excellent. The most important change in the new edition is the rewriting of the chapter on bronchiectasis; the outstanding importance of pulmonary collapse in the genesis of bronchiectasis is emphasised, and the mechanism of the process discussed. It is good to see this complete revision of clinical, and mostly stupid, theories of bronchiectasis. The new edition of the book contains obvious necessary additions in the chapter dealing with the treatment of pneumonia. Modern treatment with sulphapyridine is fully discussed and a timely word of warning issued on the subject of agranulocytosis. The book is intended for the use of those who have a special interest in pulmonary disease; nevertheless, it is a useful book of reference for all. Few problems of practice are omitted and treatment is discussed in detail.

**Insect Pests.** By WM. CLUNIE HARVEY, M.D., D.P.H., M.R. San. I., AND HARRY HILL, M.R. San. I. Pp. ix., 292. Illustrated. London: H. K. Lewis & Co. Ltd. 1940. Price 10s. 6d.—This book is primarily written for students of Public Health and all who deal with this aspect of Medicine. In their preface the authors point out that the question of insect pest control has become very important in view of the war, especially with regard to the evacuation problem. The book is divided into two parts. The first part deals in detail with the common pests, describing how to control and destroy them. A few good illustrations are displayed. The second and major portion of the book deals with the Principles and Practice of Disinfestation. It gives a good collection of the methods available, but there is no startling innovation. The purpose of the book appears to be to give as much information in as concise a form as possible and this it accomplishes well. It should prove a useful source of reference for all authorities dealing with this side of the Public Health question.

**Textbook of Public Health.** Tenth Edition. By W. M. FRAZER, O.B.E., M.D., M.Sc., D.P.H., and C. O. STALLYBRASS, M.D., D.P.H. Pp. x., 504. Illustrated. Edinburgh: E. & S. Livingstone. 1940. Price 21s.—This is Hope's *Public Health* re-written by the new authors. The book has been re-arranged and mostly re-written and the size of the volume is largely increased. It deals fully with the subject of public health, including public health legislation, and pays particular attention to the administrative aspects. It can be thoroughly recommended to students of medicine as well as to those making a study of public health.

**Diseases of the Nervous System.** Second Edition. By W. RUSSELL BRAIN, D.M., F.R.C.P. Pp. xx., 950. Illustrated. London: Oxford University Press (Humphrey Milford). 1940. Price 30s.—A new edition of this outstanding text-book of Neurology is welcome. When it first appeared in 1933, one felt it would become almost a classic. Brought up to date it is better than ever and will hold a valuable place in contemporary neurological literature. An outstanding feature of the book is the considerable section, occupying some 200 pages, devoted to general principles of neurological diagnosis based on physiology and anatomy. This part of the book may be read and re-read with profit and pleasure. Having mastered these foundations, individual clinical problems are much simplified. A second feature of interest is the table of contents which presents a successful attempt at scientific classification of diseases of the nervous system. With advancing knowledge it becomes possible to classify diseases on an aetiological basis on an increasing scale, in place of the medley of clinical and anatomical classifications formerly in vogue. The chapter on intracranial tumours is comprehensive and well illustrated. It contains some excellent skiagrams showing the rôle of ventriculography in diagnosis. It is perhaps a pity that the subject of electroencephalography receives no more than a brief mention. References to literature are there; but a slightly fuller exposition of general principles would have been welcome. The section on virus infections is full of interest, particularly the author's opening remarks on the action of neurotropic viruses in general. In this new edition he has separated off a group of disorders under the heading: Demyelinating diseases of the nervous system. This grouping is useful, though it contains, of course, a number of diseases of unknown or uncertain aetiology. Deficiency diseases receive special attention; in particular, there is an excellent account of alcoholic neuritis. The book ends with a brief but useful summary of the psychoneuroses. Throughout the reader will find excellent diagrams and illustrations; in particular histological specimens are well reproduced. Whereas the former edition contained an excellent bibliography at the end of the book, references to literature will now be found at the end of each chapter. It is inevitable that the present edition should involve an increase in the size. It does, in fact, contain an addition of some 100 pages, but by careful pruning the author has kept the text within reasonable dimensions.

**A Text-book of X-ray Diagnosis.** Vol. III. By S. COCHRANE SHANKS, M.D., F.F.R., PETER KERLEY, M.D., M.R.C.P., F.F.R., D.M.R.E., and E. W. TWINING, M.R.C.S., M.R.C.P., F.F.R., D.M.R.E. Pp. xiv., 800. Illustrated. London: H. K. Lewis & Co. Ltd. 1939. Price £3 3s.—This book, together with its companion volumes, fills a long-felt want. The reproductions of radiographs are uniformly good, and the captions are sufficiently full to render frequent references to the letterpress unnecessary. The text itself is excellent, up to date, and invaluable for cases of differential diagnoses. The index is full, diseases with several names being indexed under each disease, so that there are no annoying cross-references. The paper is of good quality, and the type clear. The book can be confidently recommended, not only to qualified radiologists, but should also prove invaluable to those preparing for radiological examinations.

**Cancer.** By GUSTAVE ROUSSY, translated by KENNETH ELLIOTT, M.B., B.S., and JOHN DE SWIET, M.B., B.S. Pp. 212. Illustrated. London: George G. Harrap & Co. Ltd. 1940. Price 6s.—This little book of two hundred pages is designed to present the intelligent layman with an account of the nature of malignant disease and of the research work that has been done by pathologists and clinicians. In an interesting historical approach to the subject the author mentions the various theories that have been held and makes brief critical comments that are of value. No attempt has been made to avoid the use of technical terms, that would be impossible, but the general presentation is very clear. The book may be warmly recommended as a balanced account of the cancer problem.

**An X-ray Atlas of Silicosis.** By ARTHUR J. AMOR, M.D., M.Sc. Pp. xii., 206. Illustrated. Bristol: John Wright & Sons Ltd. 1941. Price 30s.—Section 47 of the Workmen's Compensation Act of 1925 provides for the compensation of workmen disabled by Silicosis of the Lungs. Compensation is paid out of a General Trade Compensation Fund, administered either through a Mutual Trade Insurance Fund, or Society of Employers. Special Medical Boards appointed by the Home Secretary are the only medical authorities who can decide whether or not a workman is incapacitated by Silicosis and they are only allowed to find Silicosis to have arisen out of employment in certain specified industries. It is not surprising that from the trade union point of view this legislation presents grave defects, and the injustices arising from the operation of Section 47 are common knowledge. The Medical Boards do their part of the work on lines which are generally approved, but only within the limits of the present Silicosis schemes. These limits are oppressively and unscientifically narrow. Dr. Amor's book will serve to illustrate how far medical knowledge has advanced in the direction of recognizing the various forms of pneumokoniosis or dust-diseases of the lungs. His summaries of the pathogeny and clinical features of pulmonary fibrosis due to dust, especially silica and asbestos-dust are admirable and contain much that is new. His skiagrams are excellent and emphasize the difficulties, if not the impossibility, of recognizing Silicosis at a sufficiently early stage to protect the workman against progressive fibrosis. The general impression given by Dr. Amor's book is that an immediate recasting of the whole machinery for dealing with dust diseases of the lungs is one of the most urgent amendments which the Workmen's Compensation Acts require. In some high medical quarters the plan of the "Silicosis Boards" is regarded as the model scheme for all workmen's compensation. As a fact there is no part of the Workmen's Compensation Acts which inflicts more unjustifiable hardships. Some remedy is long overdue, and Dr. Amor's book helps to furnish evidence of the short-comings of the present scheme.

**Treatment in General Practice. Vol. IV. Surgery.** First Edition. Pp. x., 562. Illustrated. London: H. K. Lewis & Co. Ltd. 1940. Price 16s.—This volume is the last of a series in the *British Medical Journal.* It treats of things occurring indeed in general practice but many of them passing into the hands of the fully-equipped surgeon. Such here described and illustrated are the major injuries of spine or thigh, of cartilage and articulation, infected wounds of joints, complications of the crushed chest. Besides these are considered the common fractures and dislocations, infections of finger, of hand, of foot, and various other lesions of the skeletal system—flaws of the newborn, faults of the growing, atrophies of the palsied—to these every man should bring his own competence. Of the fifty-six chapters each has its interest and will find some responsive reader. Amongst things ancillary are chemotherapy, manipulative surgery, after-care in injury, bed-sores and reliefs for stumps and hernias. With novocain, as appears from place to place, one pair of hands can do much : in finger amputation, nail deformity, anal fissure, painful shoulder, catheterisation. Incise the suppurating breast not too early, nor too deeply; remove innocent tumours without mutilating. Beware of injecting thrombophiliacs ; any solution for varix will serve in hydrocele. The sections on urinary, prostatic, and rectal disorders—daily calling for common skill—display here the judgment to be expected from their authors. Mr. A. Lawrence Abel describes from his own suffering and relief an entity he thinks too seldom recognised, sacro-iliac strain. In the trail of the raid, the editor forecasts new exertions for the surgeons: he does well in gathering from the past of yesterday, to guide and to incite the craftmanship of to-morrow.

**Groves and Brickdale's Textbook for Nurses.** Sixth Edition. By E. W. HEY GROVES, M.D., D.Sc., M.S., F.R.C.S., and J. A. NIXON, C.M.G., M.D., F.R.C.P. Pp. xxx., 682. Illustrated. London: Oxford University Press (Humphrey Milford). 1940. Price 21s.—The object of this text-book for nurses is to give the modern nurse an all-round insight into the various branches of her profession—Anatomy and Physiology, Surgery, Medicine, Hygiene, Nutrition and Dietetics, with a special section on war gases. The various sections are well arranged, the illustrations specially good, the index of contents and illustrations most helpful, and the printing excellent. The book will certainly appeal to the trained nurse as a good all-round reference volume. It is well known in the profession, and this new edition will be welcomed. The Anatomy and Physiology are too advanced for the junior nurse, the book is weighty, and the price rather beyond the present-day probationer.

